# Delayed second dose of oral cholera vaccine administered before high-risk period for cholera transmission: Cholera control strategy in Lusaka, 2016

**DOI:** 10.1371/journal.pone.0219040

**Published:** 2019-08-30

**Authors:** Eva Ferreras, Belem Matapo, Elizabeth Chizema-Kawesha, Orbrie Chewe, Hannah Mzyece, Alexandre Blake, Loveness Moonde, Gideon Zulu, Marc Poncin, Nyambe Sinyange, Nancy Kasese-Chanda, Caroline Phiri, Kennedy Malama, Victor Mukonka, Sandra Cohuet, Florent Uzzeni, Iza Ciglenecki, M. Carolina Danovaro-Holliday, Francisco J. Luquero, Lorenzo Pezzoli

**Affiliations:** 1 World Health Organization, Lusaka, Zambia; 2 Epicentre, Paris, France; 3 Ministry of Health, Lusaka, Zambia; 4 Zambia National Public Health Institute, Lusaka, Zambia; 5 Médecins Sans Frontières, Geneva, Switzerland; 6 World Health Organization, Geneva, Switzerland; 7 Department of International Health, Johns Hopkins Bloomberg School of Public Health, Baltimore, Maryland, United States of America; University of New South Wales, AUSTRALIA

## Abstract

**Background:**

In April 2016, an emergency vaccination campaign using one dose of Oral Cholera Vaccine (OCV) was organized in response to a cholera outbreak that started in Lusaka in February 2016. In December 2016, a second round of vaccination was conducted, with the objective of increasing the duration of protection, before the high-risk period for cholera transmission. We assessed vaccination coverage for the first and second rounds of the OCV campaign.

**Methods:**

Vaccination coverage was estimated after each round from a sample selected from targeted-areas for vaccination using a cross-sectional survey in to establish the vaccination status of the individuals recruited. The study population included all individuals older than 12 months residing in the areas targeted for vaccination. We interviewed 505 randomly selected individuals after the first round and 442 after the second round. Vaccination status was ascertained either by vaccination card or verbal reporting. Households were selected using spatial random sampling.

**Results:**

The vaccination coverage with two doses was 58.1% (25/43; 95%CI: 42.1–72.9) in children 1–5 years old, 59.5% (69/116; 95%CI: 49.9–68.5) in children 5–15 years old and 19.9% (56/281; 95%CI: 15.4–25.1) in adults above 15 years old. The overall dropout rate was 10.9% (95%CI: 8.1–14.1). Overall, 69.9% (n = 309/442; 95%CI: 65.4–74.1) reported to have received at least one OCV dose.

**Conclusions:**

The areas at highest risk of suffering cholera outbreaks were targeted for vaccination obtaining relatively high vaccine coverage after each round. However, the long delay between doses in areas subject to considerable population movement resulted in many individuals receiving only one OCV dose. Additional vaccination campaigns may be required to sustain protection over time in case of persistence of risk. Further evidence is needed to establish a maximum optimal interval time of a delayed second dose and variations in different settings.

## Introduction

Cholera remains a significant public health problem in many parts of the world. In 2016, 38 countries reported a total of 132 121 cases including 2420 deaths, resulting in an overall case fatality rate (CFR) of 1.8% [1]. In Zambia, cholera has caused regular widespread epidemics during the rainy season since 1990. Annual epidemics have been reported between 2003 and 2010, mainly affecting the capital, Lusaka. No epidemics had been notified since 2011 [2,3], but a new cholera outbreak started in Lusaka in February 2016.

**Fig 1:**
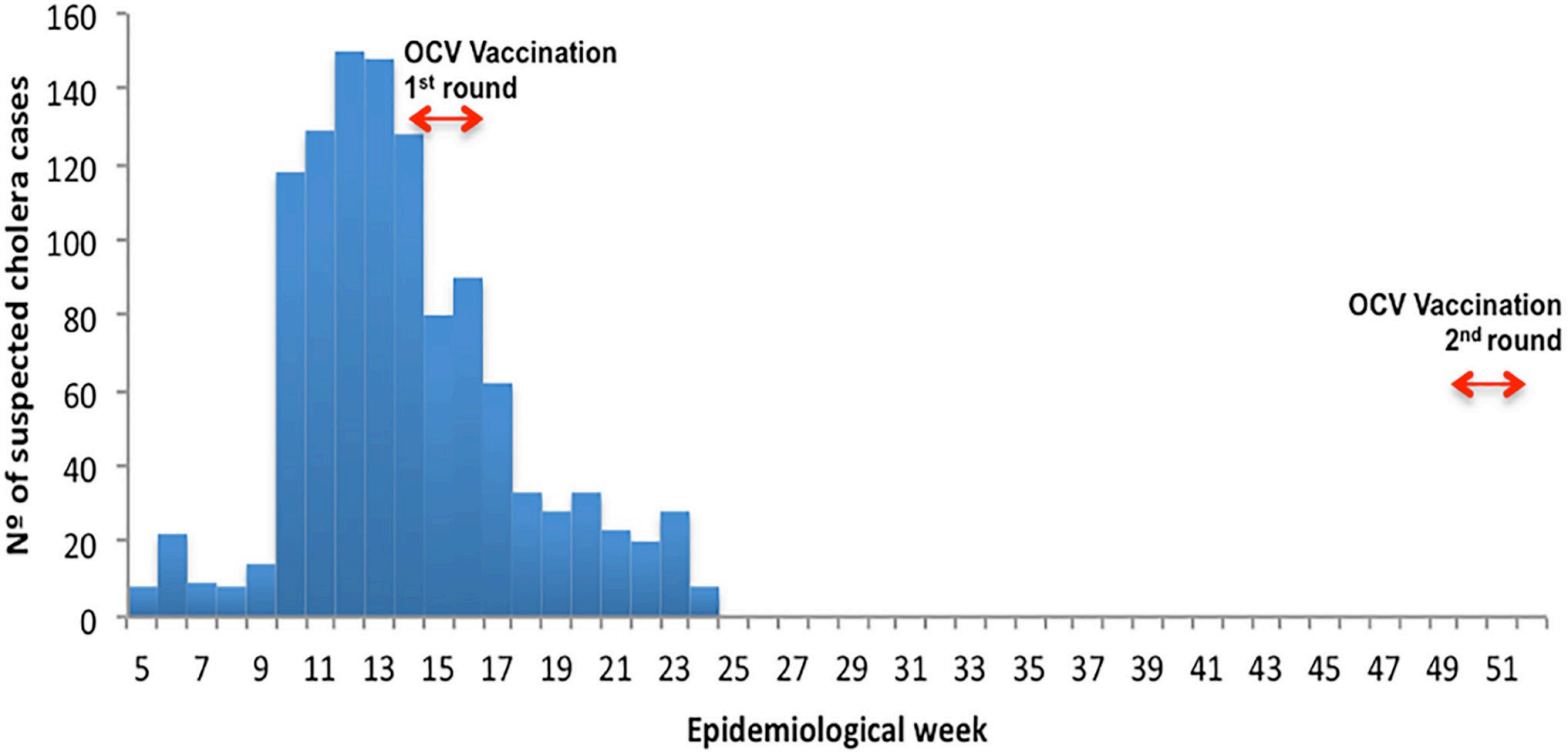
Cholera epidemic curve, Lusaka, Zambia, 2016.

In April 2016, an emergency reactive vaccination campaign using Oral Cholera Vaccine (OCV) was organized in response to the epidemic [4,5] (Fig 1). In order to halt transmission within Lusaka, and to limit the probability of spread within the country, Zambia’s Ministry of Health, Médecins Sans Frontières and the World Health Organization (WHO) decided to use a single dose of OCV, based on recent data suggesting that one dose can provide similar short-term protection (estimated to be at least 6 months) compared to two doses [6–9], since there were not enough vaccines in the global stockpile to provide two doses, two weeks apart, to the population living in areas at high risk of transmission [6,8–11].

The aim of this campaign was to vaccinate the population living in areas within Lusaka at high risk of cholera to halt transmission within the city and to limit the probability of spread within the country [12].

**Fig 2:**
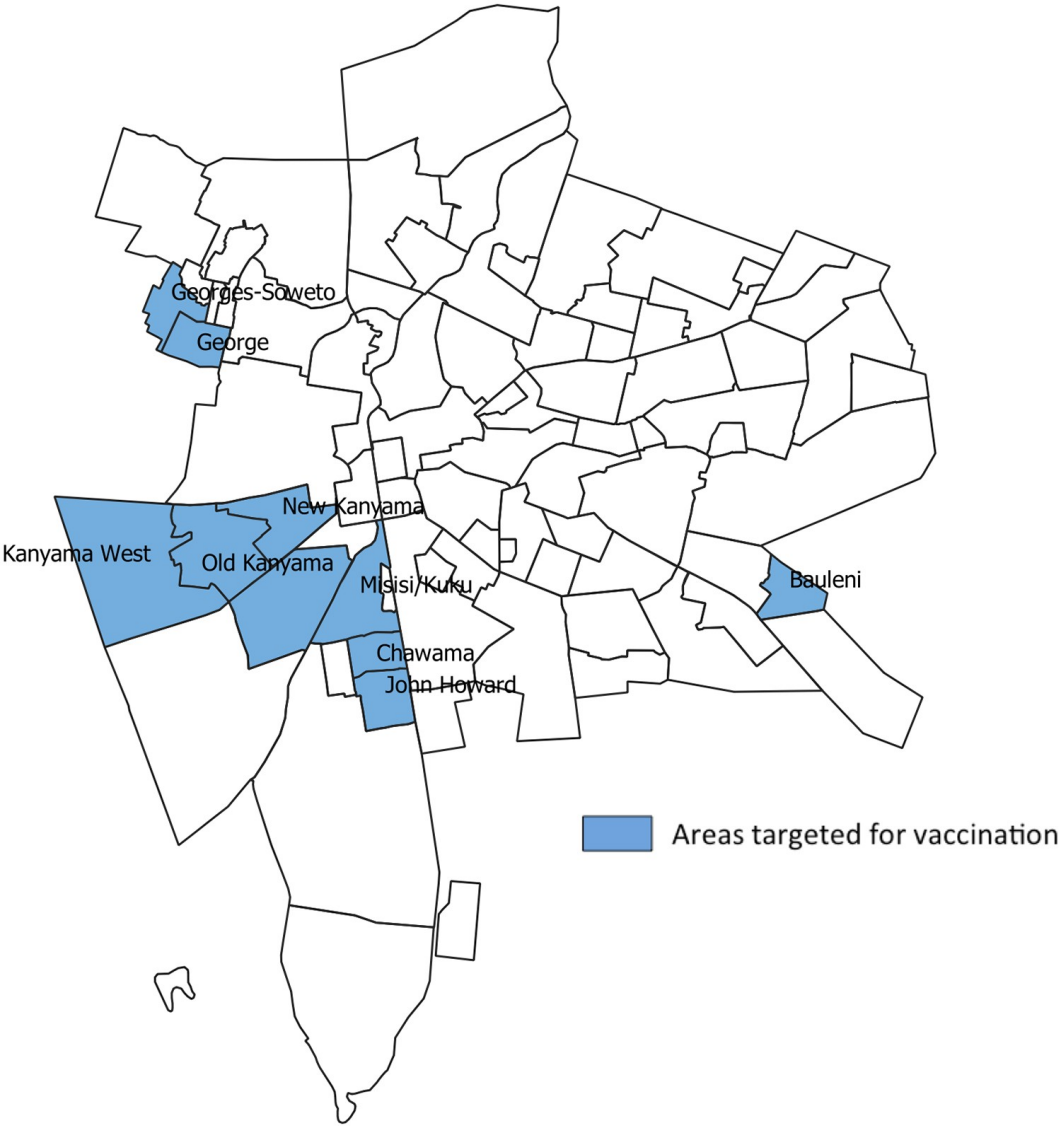
Selected areas for vaccination, Zambia, 2016.

The first OCV vaccination campaign (referred to, in this paper, as “first round”) was conducted during from 9 to 23 April 2016 [5]. Four target locations were initially selected within Lusaka district, based on either cholera attack rates or as areas historically known to be prone to cholera: 1) Bauleni, 2) Kanyama (including John Laing, New Kanyama, Old Kanyama and Kanyama West), 3) George (including George Soweto) and 4) Chawama (including John Howard and Misisi-Kuku) (Fig 2).

A total of 578 043 individuals aged 1 year or above were targeted and 424 100 OCV doses were administered, resulting in 73.4% administrative coverage [5]. The outbreak was declared officially over on 15 June 2016 with a total of 1,139 suspected cholera cases and 10 deaths reported in health structures (CFR = 0.88) [5].

From 16 to 24 December 2016, the country conducted a second round of OCV vaccination (referred to as “second round”) with the objective of increasing the duration of vaccine protection among individuals receiving the second dose and, in general, increasing the vaccinated population with any OCV dose before the rainy season (often associated with cholera). The target population was the same as for the first round. A total of 437 143 doses were administered resulting in 80.4% administrative coverage [13].

We estimated vaccination coverage after each round and the dropout rate (individuals reporting being vaccinated in April in 2016 who did not receive the second dose in December) in order to assess the proportion of people who received two OCV doses and the proportion that remained partially vaccinated or unvaccinated before the start of the 2017 cholera season in Lusaka.

## Materials and methods

### Study design

After the first round, a sample was randomly selected from the areas considered at high risk for cholera to build a cohort within vaccinated areas and non-vaccinated areas with the main objective of estimating Vaccine Effectiveness (VE) of one OCV dose [14]. The sub-cohort selected from vaccinated areas was used to estimate the vaccination coverage. After the second round, a second independent cross-sectional survey was carried out to estimate the OCV vaccination coverage.

The study population included all individuals older than 12 months residing in Bauleni, Kanyama, Chawama and George during each vaccination round. Residents were defined as persons living (sleeping and eating) for, at least, the previous two weeks in these neighbourhoods.

For the sample size calculation, we considered the following assumptions: 50% of people would receive two doses, a confidence interval of 95% (equivalent to an alpha error of 5%) and a precision of 5%. There was no need to take into account a design effect since this was a simple random sample.

We planned to visit 442 households and to interview one person per household. The representative sample of the population was selected using spatial sampling [15] in both surveys. A random point was generated in the sampling area and was retained if it coincided with a household (+/- 10 meters error); the process was repeated until the sample size was reached. One individual was randomly selected in each household among all eligible individuals being part of the household at the time of the survey. The selection of the individual at the household was done with the help of tablet device-based software. If the selected person did not meet the eligibility criteria (older than 12 months on the first day of vaccination campaign and resided in the same household in Lusaka for at least the past two weeks), the next household on the right was selected. If the selected person refused to participate, a new random point was generated.

### Data collection

Three teams comprised of 3 surveyors and 1 team leader conducted the data collection. Surveyors and team leaders were trained on the study procedures, GPS-use, sampling strategy, interviewing procedures and data collection. The questionnaire was piloted at the end of the training.

Teams conducted face-to-face interviews after obtaining written consent. A second visit was organized later in the day or on the second day if the selected participant was absent. If during the second visit the household members could not be found or refused to participate, that household was skipped. Household visits, absent households and refusals were recorded.

A standardized questionnaire was used to collect information about demographic data (age, sex and household size), having received a card and card availability at the time of the survey, vaccination status (self-reported and card-confirmed), reasons for non-vaccination (open question) and acceptability data (adverse events, taste and beliefs about the vaccine) using Kobo Toolbox software 1.4.8 (Cambridge, MA, USA).

### Data analysis

OCV coverage of two doses (full course), with at least one dose, and single dose coverage (after the first and the second round), were calculated by dividing the number of individuals reporting being vaccinated by the survey population and expressed as a percentage. Vaccination coverage included both card-confirmed and verbally reported vaccination status. We estimated 95% confidence intervals (Fisher’s exact test).

In addition, we calculated the dropout rate between rounds as follows: Dropout rate (%) = (OCV first dose—OCV second dose/ OCV first dose) x 100.

Data analysis was carried out using R statistical software, version 3.2.3, taking into account the sampling scheme.

### Ethical considerations

The Ethical Review Boards of the University of Zambia and the Johns Hopkins Bloomberg School of Public Health (USA) approved the study protocol for the cohort recruitment.

The study protocol for the coverage survey was approved by the ERES-Converge-Ethical Review Committee in Zambia and the clearance to conduct the survey was granted by the National Health Research Agency.

Privacy, confidentiality and rights of participants were guaranteed during and after the study. A signed informed consent was sought from each individual, or in case of a minor, from their guardian. From the 947 participants in both rounds, 417 were minors. All data was entered and analysed anonymously.

## Results

### Sample description

From 17 April 2016 to 25 May 2016, following the first round of the OCV campaign, 505 individuals were recruited from vaccinated areas in Lusaka as part of the survey cohort. The median age was 20 years old (interquartile rage (IQR): 9–30 years). The participation of males (59%) was more than that of women. The average household size was 5 (standard deviation: 2.3). The participation rate was: 100%

From 3 to 17 March 2017, 478 households were visited for the vaccination coverage survey conducted after the second round,. Occupants from 11 households could not be found after two visits; 467 individuals were screened for participation in the survey. Among these individuals, 18 refused to participate and 7 were ineligible.

Finally, 442 were included in the analysis. The average household size was 4 (standard deviation: 2.4). The median age was 21 years old (IQR: 10–34 years). The male/female ratio was 0.64.

### First vaccination round: April 2016

Overall, in the survey conducted in May 2016, 68.5% (346/505; 95%CI: 64.3–72.5) of the target population reported having received one OCV dose during the first round in April 2016 (Table 1). The percentage of individuals having the vaccination card among those reporting being vaccinated in the first round was 50.9%.

**Table 1:** Vaccination coverage for two, one and at least one OCV dose, and for the first and the second round of the campaign; by age group, sex and township.

	2 doses VC		1 dose VC		At least 1 dose VC		1st round VC		1st round VC*		2nd round VC	
	n	% (95%CI)	n	% (95%CI)	N	% (95%CI)	n	% (95%CI)	n	% (95%CI)	n	% (95%CI)
**Overall VC**	150	33.9 (29.5–38.6)	159	36.0 (31.5–40.6)	309	69.9 (65.4–74.1)	346	68.5 (64.3–72.5)	198	44.8 (40.0–49.6)	261	59.0 (54.3–63.7)
**1 to 5 years**	25	58.1 (42.1–72.9)	13	30.2 (17.2–46.1)	38	88.4 (74.9–96.1)	51	85.0 (73.4–92.9)	27	62.8 (46.7–77.0)	36	83.7 (69.3–93.2)
**5 to 15 years**	69	59.5 (49.9–68.5)	38	32.7 (24.3–42.1)	107	92.2 (85.8–96.4)	116	81.1 (73.7–87.2)	79	68.1 (58.8–76.4)	97	83.6 (75.6–89.8)
**15 + years**	56	19.9 (15.4–25.1)	108	38.4 (32.7–44.4)	164	58.4 (52.4–64.2)	179	59.3 (53.5–64.9)	92	32.7 (27.3–38.6)	128	45.5 (39.6–51.6)
**Males**	54	31.4 (24.5–38.9)	52	30.2 (23.5–37.7)	106	61.6 (53.9–68.9)	117	62.2 (54.9–69.2)	66	38.4 (31.1–46.1)	94	54.6 (46.9–62.2)
**Females**	96	35.8 (30.1–41.9)	107	40.0 (34.0–46.1)	268	75.7 (70.2–80.7)	229	72.2 (67.0–77.1)	132	49.2 (43.1–55.4)	167	62.3 (56.2–68.1)
**Bauleni**	6	27.3 (10.7–50.2)	9	41 (20.7–63.6)	15	68.2 (45.1–86.1)	20	80.0 (59.3–93.2)	7	31.8 (13.9–54.9)	14	63.6 (40.6–82.8)
**Chawama**	23	35.4 (23.9–48.2)	26	40 (28–52.9)	49	75.4 (63.1–85.2)	51	68.0 (56.2–78.3)	27	41.5 (29.4–54.4)	45	69.2 (56.5–80.1)
**George**	24	38.1 (26.1–51.2)	23	36.5 (24.7–49.6)	47	74.6 (62.1–84.7)	62	87.3 (77.3–94.0)	36	57.1 (44.09–69.5)	35	55.6 (42.5–68.1)
**George-Soweto**	6	31.6 (12.6–56.5)	9	47.4 (24.4–71.1)	15	78.9 (54.4–93.9)	15	71.4 (47.8–88.7)	8	42.1 (20.2–66.5)	13	68.4 (43.4–87.4)
**John-Howard**	10	41.7 (22.1–63.4)	9	37.5 (18.8–59.4)	19	79.2 (57.8–92.9)	16	55.2 (35.7–73.6)	10	41.7 (22.1–63.3)	19	79.2 (57.8–92.9)
**Jhon Laing**	14	30.4 (17.7–45.7)	20	43.5 (28.9–58.9)	34	73.9 (58.9–85.7)	27	50.0 (36.1–63.9)	19	41.3 (26.9–56.8)	29	63.0 (47.5–76.8)
**Kanyama West**	13	27.6 (15.6–42.6)	15	31.9 (19.1–47.1)	28	59.6 (44.3–73.6)	29	56.9 (42.2–70.7)	18	38.3 (24.5–53.6)	23	48.9 (34.1-.63.9)
**Misisi-Kuku**	21	37.5 (24.9–51.4)	19	33.9 (21.8–47.8)	40	71.4 (57.8–82.7)	44	69.8 (57.0–80.8)	27	48.2 (34.6–61.9)	34	60.7 (46.7–73.5)
**New Kanyama**	6	23.1 (8.97–43.6)	6	23.1 (8.9–43.6)	12	46.1 (26.6–66.6)	20	69.0 (49.2–84.7)	10	38.5 (20.2–59.4)	8	30.8 (14.3–51.8)
**Old Kanyana**	27	37.5 (26.48–49.7)	23	31.9 (21.4–43.9)	50	69.4 (57.45–79.8)	62	71.3 (60.6–80.5)	36	50.0 (37.9–62)	41	56.9 (44.7–68.6)

*This estimate represents the percentage of people living in the targeted areas during March 2017 survey who verbally reported having been vaccinated in April 2016.

During the coverage survey following the second OCV dose, only 44.8% (198/442; 95%CI: 40.0–49.6) of people living in the targeted areas reported having been vaccinated in April 2016. When we stratified by age groups, we found that for 5 to 15 years the coverage was 68.1% (79/116; 95%CI: 58.8–76.4) and 32.7% (92/281; 95%CI: 27.3–38.6) for adults. (Table 1).

### Second vaccination round: December 2016

The study estimated that 59.0% (261/442; 95%CI: 54.3–63.7) of the target population received one dose of vaccine during the second OCV round in December 2016 (Table 1). The percentage of individuals presenting the vaccination card among those reporting having been vaccinated was 24.0% (n = 83).

### Overall two dose and single dose vaccination coverage

Coverage with two OCV doses was 33.9% (150/442; 95%CI: 29.5–38.6) and 36.0% reported having received a single OCV dose (159/442; 95%CI: 31.5–40.6). Hence, at the time of the 2017 vaccination coverage survey, 69.9% (309/442; 95%CI: 65.4–74.1) reported to have received at least one OCV dose, in either of the two rounds, (Table 1). The dropout rate was 10.9% (95%CI: 8.1–14.1).

### Stratified analysis by age, sex and township

Table 1 shows the overall vaccination coverage with two OCV doses, with single OCV dose and the coverage achieved in the first and second vaccination rounds stratified by age group, sex and township.

Vaccination coverage with two OCV doses was higher among children ((58.1% (25/43; 95%CI: 42.1–72.9) for children 1–5 years old and 59.5% (69/116; 95%CI: 49.9–68.5) for 5–15 years old) than in adults (19.9% (56/281; 95%CI: 15.4–25.1) for adults above 15 years old) (Table 1).

**Fig 3:**
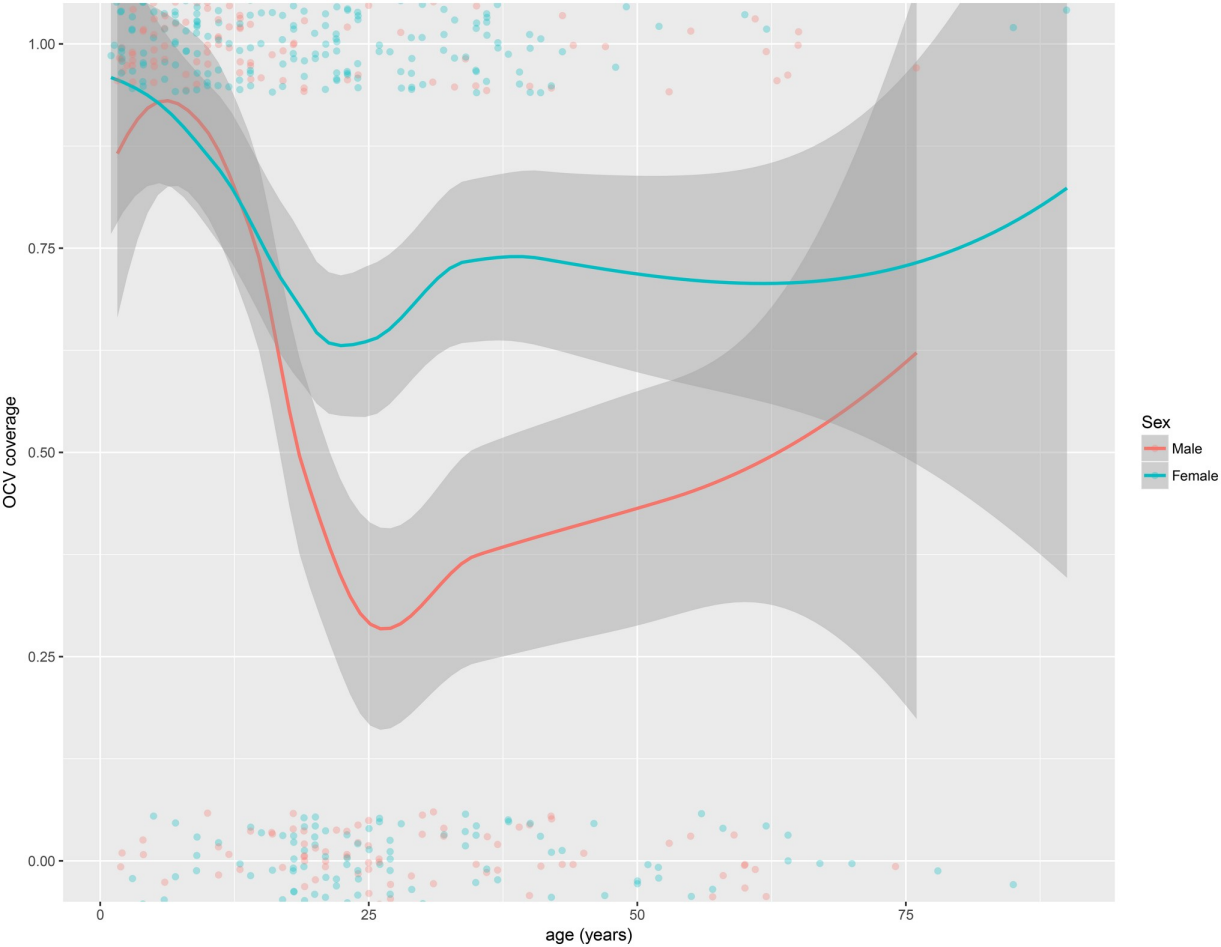
Vaccination coverage with at least one OCV dose by age and sex, Zambia, 2016.

**Fig 4:**
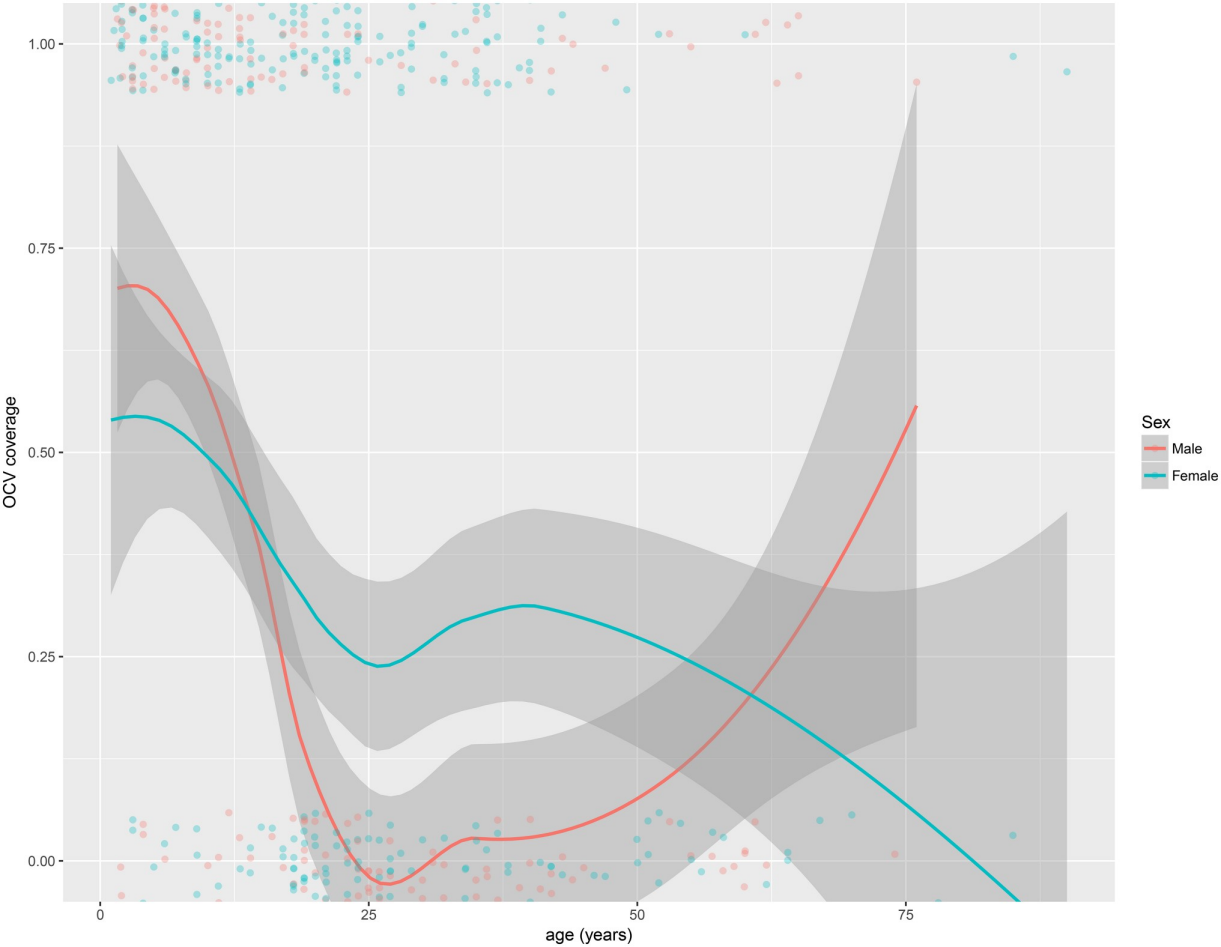
Vaccination coverage with two OCV doses by age and sex, Zambia, 2016.

The vaccination coverage with at least one OCV dose was higher among women 75.7% (95%CI: 70.2–80.7) than among men 61.6% (95%CI: 53.9–68.9) (Fig 3). This was especially true for the coverage with two doses among individuals between 25 and 50 years old (Fig 4).

The OCV coverage varied by township, with the highest vaccination coverage with at least one vaccine dose being observed in John-Howard (79.2%, 95%CI: 57.8–92.9) and the lowest in New Kanyama (46.1%, 95%CI: 26.6–66.6) (Table 1).

## Discussion

Two rounds of vaccination using OCV were organized in 2016 in Lusaka with a similar number of doses distributed in both campaigns. The vaccination coverage achieved in the second round (59.0%) in the targeted areas for vaccination was lower than the one obtained after the first vaccination round in April 2016 (68.6%). Among the 36.0% of individuals vaccinated with a single vaccine dose, the 30% received this dose in April 2016 and the 70% in December 2016. That suggest that only a fraction of the population vaccinated in April 2016 was still present in the vaccination areas in December, limiting the possibility to obtain high two OCV dose vaccination coverage.

The OCV vaccination coverage with a complete vaccine course was 33.9%. We found large differences in two-dose vaccination coverage among children (58.1% in under 5 years old and 59.5% in 5 to 15 years old children) and adults (19.9%), even if during the campaign this issue was detected and a catch-up activity was performed to narrow the gap in coverage between age groups [5]. Moreover, coverage in males was lower than in females, with this difference being higher for coverage with at least one OCV dose (61.6% in males and 75.7% in females). Several publications have already described this issue in different contexts as in Guinea, Thailand and Bangladesh [16–18].

This was the first campaign using an eight-month delayed interval between doses. Most previous campaign coverage surveys focused on measuring full vaccination (two doses) coverage immediately after the implementation of the standard two doses campaign with a two-week interval [19–21]. However, these evaluations have not addressed changing in OCV coverage over time. The survey implemented in Lusaka in March 2017 also allowed measuring the proportion of people in the target population who had been vaccinated in the April 2016 round. Despite possible information bias affecting this estimate, this percentage was relatively low, especially among the adult population, and raises an important question about the need to re-vaccinate populations to maintain a certain level of OCV coverage over time. In this study, coverage with two OCV doses (33.9%) was lower than expected. The population mobility in these urban compounds is high and likely related to economical, seasonal, and work factors, especially in Kanyama townships. Moreover, during the 2016 OCV vaccination campaign, there were xenophobic riots in some of the targeted townships for vaccination [22].

An aspect that could be improved in future evaluations, considering the possible mobility of the population as a partial explanation of the findings, is to include additional details about when the individuals in the survey arrived to the target areas and from where, or how long they have lived in the area in order to better characterize the mobility patterns.

The delayed second OCV round allowed achieving vaccination coverage with at least one dose to 69.9% just before the start of the 2017 cholera season in Lusaka. Recent studies have shown that a single OCV dose provides a similar protection in the short term as a two-dose schedule [6–8,14]. Therefore, the delayed second OCV dose might have been an effective way to achieve a good level of protection before the cholera season in Lusaka and prevent or mitigate the possibility of a new outbreak in the vaccinated areas during 2017.

The desired sample size was achieved, which allowed having adequate precision for the overall estimates. However, the precision of the estimates by township, especially in Bauleni, was not optimal and stratified sampling by smaller geographical units should be considered in the future considering the differences observed by township.

The major limitation of this study was the high percentage of vaccinated individuals with missing vaccination cards despite the short interval between the vaccination campaign (April 2016 and December 2016) and the coverage surveys (May 2016 and March 2017). For the first vaccination round, this situation was likely affected by the poor-quality of the paper used for card printing. Vaccination cards ran out in the second round. Although it was recommended that people should bring their vaccination cards from the previous round, several people reported having forgotten or misplaced their cards. Plain pieces of paper were issued as proof of vaccination when the vaccination cards ran out. This could be an additional reason for the low card retention. The short delay between the campaign and the survey recruitment may have controlled the risk of having a large information bias in the vaccination status; however, it still highlights the difficulty to be certain about the vaccination coverage achieved. Also, possible recall or reporting bias may have been at play due to the 11-month delay between the first round and the March 2017 survey, which could have led to underestimating the coverage with two doses. It is likely that the people who did not take the OCV dose in December 2016 would have had more difficulties to recall having received the April OCV dose. This may explain the big discrepancies in the coverage of the first round among adults (and especially among males) between the 2016 and 2017 surveys (41.3% of coverage among males in the 2016 survey and 14,7% in the 2017 survey).

## Conclusions

The areas at highest risk of suffering cholera outbreaks were targeted for a vaccination campaign obtaining relatively high vaccine coverage (68.5% in April and 59% in December 2016). Overall, 69.9% of the people living in these areas, by March 2017, reported having received at least one dose of OCV. Considering the good short-term effectiveness of one vaccine dose, this might have been adequate to avoid a large outbreak in these high-risk areas during the 2017 main cholera season (January to June). On 6 October 2017 a new cholera outbreak was declared, having the index case in Chipata, an area not targeted during the 2016 OCV campaign.

However, the high population mobility suggests that coverage might drop over time, in particular among adults, where vaccination coverage levels are lowest (38.4% with one dose, 58.4% with at least one dose and 19.9% with two doses). This issue should be evaluated and re-vaccination of these areas may be required to maintain good levels of protection. Furthermore, specific strategies may need to be developed to better reach the adult population, especially males, in order to increase OCV coverage among those aged 15 years and more.

We can conclude that, a strategy that includes a delayed second dose is pertinent in a scenario where it is not possible to vaccinate with two OCV doses in a normal two-week schedule, such as that of an epidemic situation where a prompt and reactive response is necessary and there is a limited stockpile of vaccines. This strategy allows to urgently control the outbreak with an increased number of doses (in terms of reducing morbidity, mortality and transmission) and, in addition, to increase the vaccine protection in the longer term. This example from Lusaka also suggests that for highly mobile populations, where the proportion of persons vaccinated following a mass vaccination campaigns might drop over time, a delay of eight months between OCV doses might be too long to ensure that the target population receives the recommended two doses. On the other hand, annual OCV campaigns before the cholera season might increase the number of individuals covered with at least one OCV dose and, consequently, might be also an effective strategy to reduce the risk of outbreaks in places at high risk of cholera transmission.

## Supporting information

S1 Dataset (XLSX)

S2 Dataset (XLSX)

S1 File (XLSX)
